# DDX11L: a novel transcript family emerging from human subtelomeric regions

**DOI:** 10.1186/1471-2164-10-250

**Published:** 2009-05-28

**Authors:** Valerio Costa, Amelia Casamassimi, Roberta Roberto, Fernando Gianfrancesco, Maria R Matarazzo, Michele D'Urso, Maurizio D'Esposito, Mariano Rocchi, Alfredo Ciccodicola

**Affiliations:** 1 Institute of Genetics and Biophysics "A. Buzzati-Traverso" (IGB), CNR, 80131 Naples, Italy; 2 Current address: Department of General Pathology, Second University of Naples, 80138 Naples, Italy; 3 Department of Genetics and Microbiology, University of Bari, 70126 Bari, Italy

## Abstract

**Background:**

The subtelomeric regions of human chromosomes exhibit an extraordinary plasticity. To date, due to the high GC content and to the presence of telomeric repeats, the subtelomeric sequences are underrepresented in the genomic libraries and consequently their sequences are incomplete in the finished human genome sequence, and still much remains to be learned about subtelomere organization, evolution and function. Indeed, only in recent years, several studies have disclosed, within human subtelomeres, novel gene family members.

**Results:**

During a project aimed to analyze genes located in the telomeric region of the long arm of the human X chromosome, we have identified a novel transcript family, *DDX11L*, members of which map to 1pter, 2q13/14.1, 2qter, 3qter, 6pter, 9pter/9qter, 11pter, 12pter, 15qter, 16pter, 17pter, 19pter, 20pter/20qter, Xpter/Xqter and Yqter. Furthermore, we partially sequenced the underrepresented subtelomeres of human chromosomes showing a common evolutionary origin.

**Conclusion:**

Our data indicate that an ancestral gene, originated as a rearranged portion of the primate *DDX11 *gene, and propagated along many subtelomeric locations, is emerging within subtelomeres of human chromosomes, defining a novel gene family. These findings support the possibility that the high plasticity of these regions, sites of DNA exchange among different chromosomes, could trigger the emergence of new genes.

## Background

Human subtelomeric sequences are extraordinarily dynamic and variable regions near the ends of chromosomes, and represent the transition sites between chromosomes-specific sequences and telomeric repeats capping each chromosomal end [1]. The unusual nucleotide composition of human subtelomeres was first evident from fluorescence *in situ *hybridization (FISH) analysis of cloned segments of subtelomeric regions [2].

However, to date, the large variability of subtelomeric sequences is underrepresented in the complete human genome database, because of the low representation of clones covering the proximity of these regions in the libraries used in the Human Genome Project. Moreover, the high level of polymorphism found in the human subtelomeres, makes assembling multiple different alleles of the same chromosome more challenging than most of the regions of human genome.

The comparative analysis of fully sequenced subtelomeres of human 4p, 16p, 22q, Xq and Yq, has revealed a common structure, in which the proximal and distal subtelomeric domains are separated by a stretch of degenerate TTAGGG repeats [3-6]. The subtelomeric repeats identified in these studies, often show a polymorphic chromosomal distribution, due to infrequent events of non-homologous recombination that transfer irregular DNA patches to some chromosomes but not to others [7]. Furthermore, the high rate of homology shared by some subtelomeric and centromeric sequences, indicates past transfers of genomic material among these sites [8].

The analysis of subtelomeric sequences has shown that these fragments are more than simple functionless DNA spacer regions joining the telomere to chromosome-specific sequences [9]. Indeed, subtelomeres seem to be involved in various processes such as homologous and non-homologous chromosome recombination events, and also in the telomere healing, consisting in telomere elongation in the absence of telomerase [10,11]. It has also been postulated that subtelomeres attenuate the telomere position effect (TPE), a gene-silencing phenomenon due to the heterochromatic state of telomeres involving all the genes located in the proximity of the telomeres [12]. Furthermore, in addition to their structural role, the subtelomeric regions of human chromosomes contain many genes, members of several gene families [13,14].

During the evolution of genomes, a huge number of new genes have been created by gene-duplication events [14]. These processes are most commonly followed by one or more mutational events that silence one member of the pair, although they may alternatively undergo processes of sub-functionalization or neo-functionalization. In the former, both the pair members acquire degenerative mutations reducing their pattern of splicing and their activity compared to the single ancestral gene. The latter, conversely, contributes to the creation of a gene family with novel functions, as one gene accumulating mutations may acquire a different function, while the other member still retains the original one [15,16].

In this paper we describe the characterization of a novel multicopy transcript closely adjacent to TelBam3.4 derived sequences. Previously reported as a pseudogene of *CHLR1*-related helicase gene [5,17,18], the newly defined transcript family, *DDX11L*, was disclosed in the subtelomeres of various human chromosomes. The identification of this novel gene family and its spreading along the primate lineage, offers novel insights into our understanding of subtelomeres dynamics, and into the emergence of a multicopy transcript from an inactive pseudogene.

Although we have not yet defined a biological function for the newly identified transcript family, we found that these genomic regions are actively transcribed (in many human tissues) and also undergo canonical splicing. To date, we cannot define these transcriptionally active regions as functional genes, but we cannot definitely exclude it. In the paper the term "gene family" will be referred to "transcriptionally active regions family".

## Results

### Genomic structure and localization of DDX11L genes

Our group has contributed to the full sequence project of human X chromosome [19]. In our previous experiments [5], we sequenced 400 kb of the subtelomeric region of the long arm of human X chromosome (Xq-PAR) and the related telomere sequence. During this work, the bioinformatics analysis revealed, within this genomic region, the presence of 4 genes (*SYBL1, HSPRY3, IL9R *and *CXYorf1*, currently named *WASH*) and 2 pseudogenes (AMD2p and CHL1p).

A routine comparison of the previously identified CHL1p pseudogene sequence with the available databases revealed that this sequence shares high homology with the sequence corresponding to *CHLR1*-related helicase gene (*DDX11 *gene), mapping to 12p11 and 12p13, as well as with human TelBam 3.4, telomere-associated sequence [2,17].

**Figure 1:**
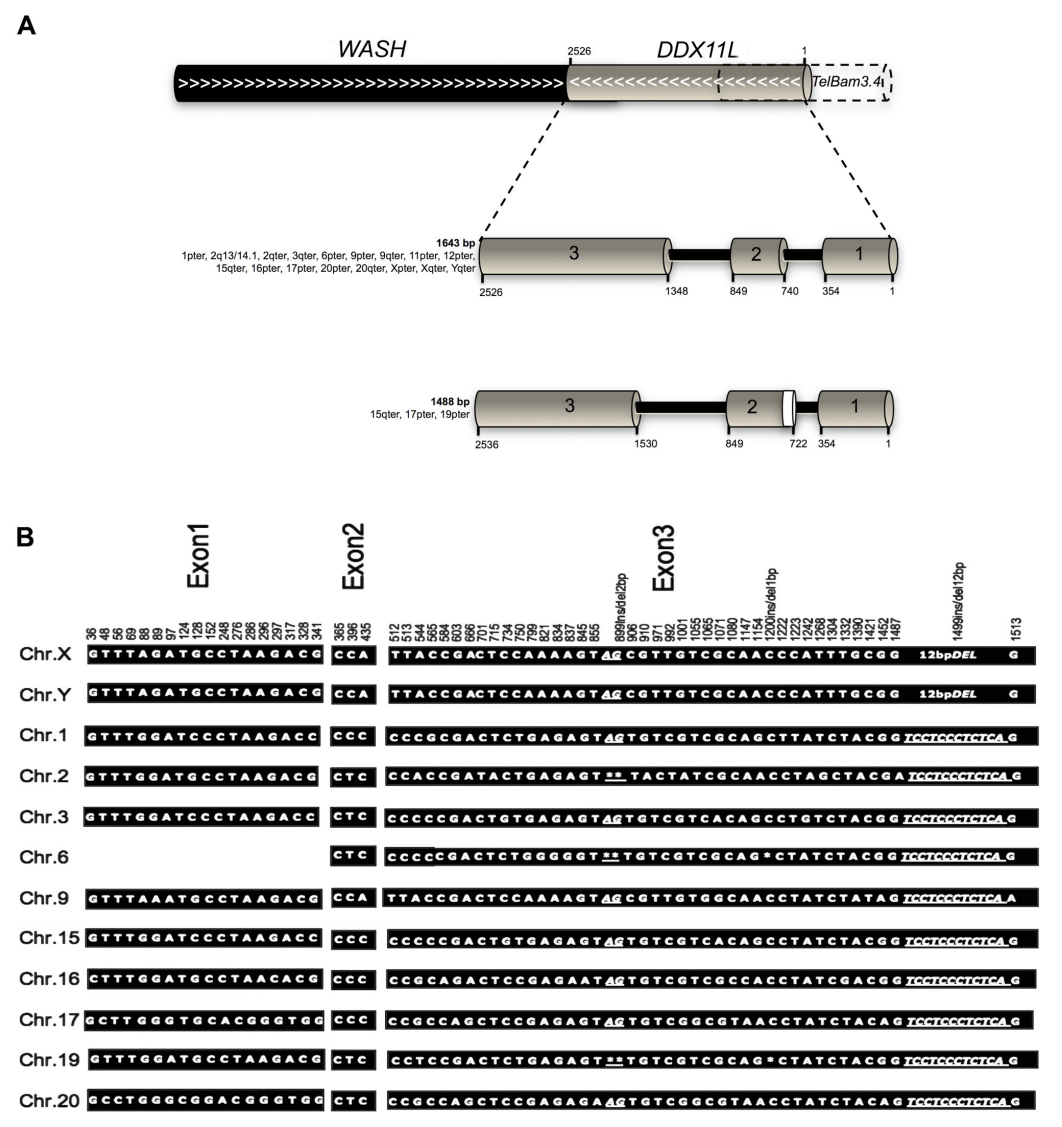
**Schematic representation and *intra*-exon nucleotide variations of *DDX11L *gene family members**. (A) On the top. *DDX11L *genomic localization between *WASH *genes and the telomeric repeats. White arrows indicate the sense of gene transcription. Grey boxes represent the exons of *DDX11L *genes. Numbers indicate the exon boundaries. On the bottom. *DDX11L *alternative spliced transcript of 1481 bp, in the chromosomes 15, 17 and 19. ATG is located in position 317 and the TAG stop codon in position 718. This transcript shows a significant ORF of 402 bp, encoding a putative protein of 133 aa. (B) Positions of nucleotide variations referred to X chromosome cDNA. Asterisks and underlined nucleotides represent deletion/insertion sites.

A total of 14 spliced ESTs (AA663731, AI418732, AI468499, AI908236, AI908251, AI908255, AI969596, AW081020, BC005070, BE295252, BF197227, BF222384, BF375356, BG254338, BI909979, BM920887, and BM920886) derived from multiple tissues, with 100% of sequence identity with the telomeric region of the human Xq28, were detected. A resulting 1643 bp consensus cDNA sequence was aligned to the finished and unfinished human genomic sequences (BLASTN at NCBI) allowing us to determine the correct exon/intron structure of this novel gene, named *DDX11L16 *from the HGNC (HUGO Gene Nomenclature Committee). The entire *DDX11L16 *gene spans 2526 bp of genomic DNA and consists of three exons of 354 bp, 108 bp and 1180 bp respectively, separated by two intervening sequences of 385 bp and 498 bp (see Figure 1A). All the identified donor and acceptor splice sites sequences are in accordance with the GT-AG rule [20]. In addition, the alignment of *DDX11L16 *cDNA to the fully sequenced subtelomeres of Xq, Yq, 16p, 15q, 1p, 9p chromosomes and the pericentromeric region of human chromosome 2 (2q13/14.1) has shown the presence of other *DDX11L16 *copies on these chromosomes. The bioinformatics analysis has revealed they have the same telomere-to-centromere orientation, mapping about 400 bp proximal to telomere associated region (TAR) sequences at 5' end, while the 3' end overlaps a few bp of the 3' end of *WASH *genes [14]. Furthermore, we assessed, through PCR on DNAs from the monochromosomal somatic hybrids, the presence of this novel gene also on human 3, 6, 17, 19 and 20 chromosomes, and sequenced the *DDX11L *gene copies on each above-mentioned chromosome (accession numbers: AM992865, AM992866, AM992867, AM992868 and AM992869). In this way, we defined a novel gene family – the most telomeric ever identified – named *DDX11L*. The total number of *DDX11L *gene copies ranges from 16 to 19 in the analyzed individuals (see the FISH analysis in the "*DDX11L *genes are present in multiple copies in primates' genomes" paragraph), even though these copies were collectively found in 18 different sites (see table 1).

**Table 1:** *DDX11L *gene family

**Gene symbol**	**Localization**	**New sequence**	**FISH**	**Genebank**
*DDX11L1*	1p36.33	-	-	+

*DDX11L2*	2q13/14.1	-	+	+

*DDX11L3*	3q29	+	+	-

*DDX11L4*	6p25.3	+	+	-

*DDX11L5*	9p24.3	-	+	+

*DDX11L6*	9q34.3	-	+	-

*DDX11L7*	11p15.5	-	+	-

*DDX11L8*	12p13.33	-	+	-

*DDX11L9*	15q26.3	-	+	+

*DDX11L10*	16p13.3	-	-	+

*DDX11L11*	17p13.3	-	+	-

*DDX11L12*	19p13.3	+	+	-

*DDX11L13*	20p13	+	+	-

*DDX11L14*	20q13.3	+	+	-

*DDX11L15*	Xp11.32	-	+	-

*DDX11L16*	Xq28	-	+	+

*DDX11L16*	Yq12	-	+	+

*DDX11L17*	2q37.3	-	+	-

All the sequences of *DDX11L *gene family members – both known and sequenced during this work – were aligned. The alignment revealed that these sequences contain inter-chromosomal single base variations within exons and introns. This analysis allowed us to detect a specific haplotype for each analyzed chromosome, also revealing the presence of a 12 bp deletion in the 3' end of *DDX11L16 *gene in Xq/Yq region (see Figure 1B). The sequence analysis also revealed the presence of different open reading frames (ORF; data not shown). Furthermore, since the sequence alignment revealed that *DDX11L *genes are distal to *WASH *genes, overlapping a few bp within the fully sequenced subtelomeres of Xq, Yq, 16p, 15q, 1p, 9p and 2q13/14.1, we investigated whether these two genes also overlapped in the subtelomeres of 3, 6, 11, 17, 19 and 20 human chromosomes.

**Figure 2:**
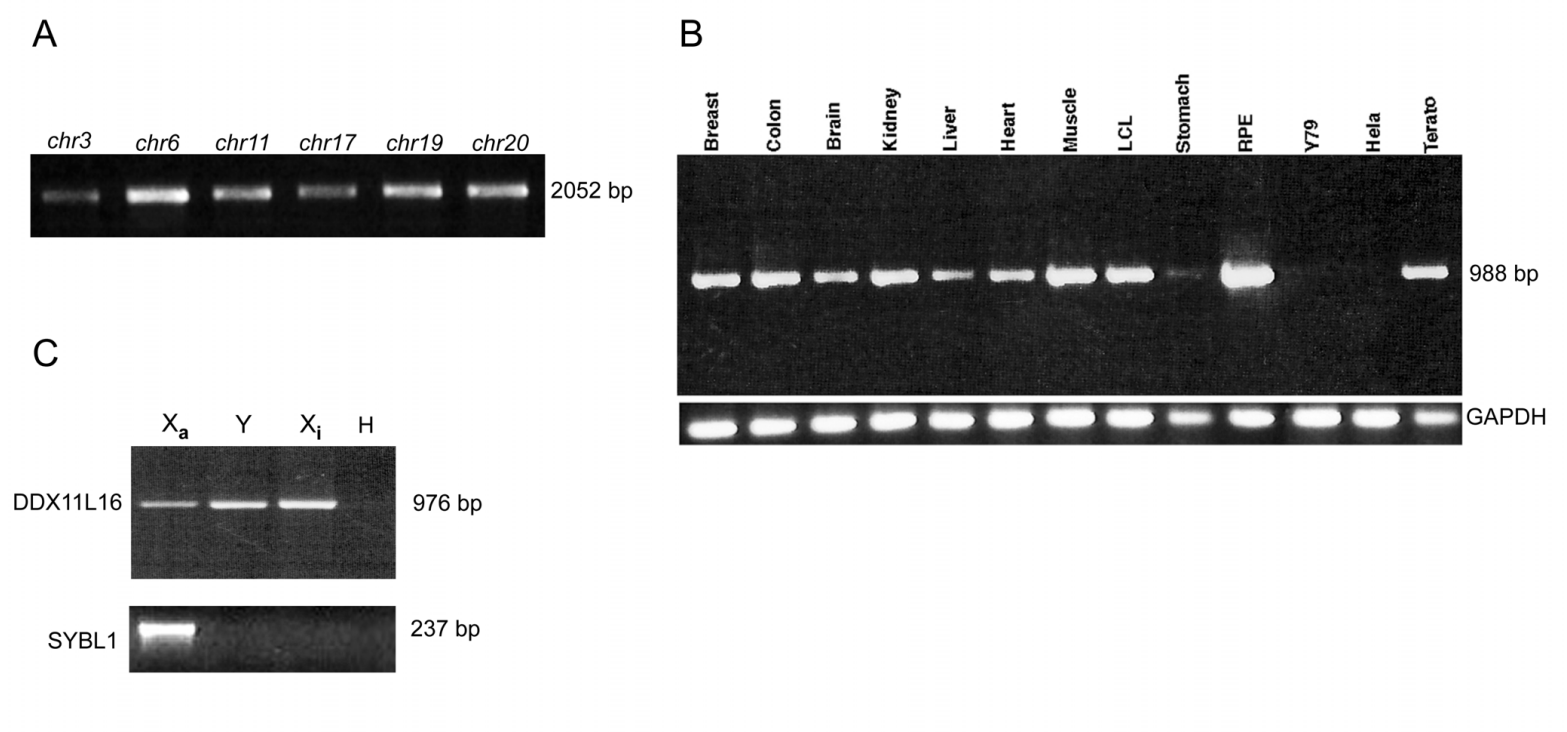
***DDX11L *gene family analysis**. (A) PCR products of 2,1 kb amplified on monochromosomal somatic hybrids. Primer pair localization as described in the section "Genomic structure and localization of DDX11L genes". (B) Expression pattern in 8 human tissues and 4 stabilized tumoral cell lines. RPE (retinal pigmentum epithelium); Y79 (retinoblastoma cell line); LCL (lymphoblastoid cell line with 5 X chromosomes)(C) X inactivation pattern on monochromosomal hybrid cell lines containing X_a_, X_i _and Y human chromosomes.

To this purpose, we performed PCR amplifications on DNA from the monochromosomal somatic hybrids, using a primer in the *WASH *genes coding region and the other within *DDX11L *genes. Thus, by direct sequencing of PCR fragments, we demonstrated the genomic proximity of the newly identified *DDX11L *genes with the *WASH *gene family members [14], also located within the subtelomeres of the above-mentioned human chromosomes (Figure 2A).

### Expression analysis of *DDX11L genes*

We detected the expression of the *DDX11L *gene family members on 1, 2, 3, 6, 9, 11, 12, 15, 16, 17, 19, 20 human autosomes, using a semiquantitative RT-PCR on RNAs extracted from the monochromosomal somatic hybrid cell lines [see Additional file 1]. Expression analysis revealed the presence of an alternatively spliced transcript, a short splice variant of 1481 bp in the *DDX11L9*, *DDX11L11 and DDX11L12 *genes, on 15, 17 and 19 chromosomes, respectively (see Figure 1B; accession ns. AM992880, AM992881 and AM992878). Sequence analysis revealed that the short splice variant consists of the entire exon 1, an exon 2, 18 bp longer at the 3' end, and an exon 3, 180 bp shorter at the 5'. These newly identified donor and acceptor splice sites are in accordance with the GT-AG rule [20]. A significant ORF of 402 bp in the *DDX11L9 *short splice variant – ATG located at the position 317 and TAG at the position 718 – was found, encoding a putative protein of 133 aminoacids significantly homologous (up to 88%) to DDX11 protein. Similarly, it was found a 336 bp ORF in the *DDX11L11 *short splice variant – ATG at the position 383 and TAG at the position 718 – encoding a putative protein of 111 aminoacids. Conversely, we found a significant, but shorter, ORF of 270 bp in the *DDX11L12 *splice variant, with the ATG at the position 734 and the TAG at the position 1003. This transcript encodes a putative protein of 89 aminoacids, with a high sequence identity (up to 80%) in the N-terminal region of DDX11 protein (aminoacids 1–36). On the opposite, no significant similarity with known proteins in the C-terminal region was detected. We have, therefore, identified at least 3 potentially intact *DDX11L *variants (with different ORFs), and it is likely that some of these may have slightly different functions, although we have no evidence for positive selection in mammalian *DDX11L1 *genes. Moreover, a deeper sampling would be required to account for all identified gene copies.

Furthermore, to assess the expression pattern of the *DDX11L *genes, we carried out an RT-PCR analysis – amplifying a product of 976 bp – on RNAs derived from 8 human tissues and 4 different human cell lines (three of them from stabilized cancers). This analysis revealed that the *DDX11L *genes are ubiquitously expressed with no detectable expression only in retinoblastoma (Y79) and HeLa cell lines (see Figure 2B).

The assessment of sex – and chromosome-specific – expression was attempted using oligonucleotide primer pairs designed across a cDNA-specific sequence, in the hybrid cells employed to analyze *DDX11L16*. The gene expression was detected in three hybrids containing active X (GM06318B), inactive X (THX88) and Y (GM06317) chromosome, already used for inactivation experiments [21]. The expression analysis confirmed, as awaited for its localization in the PAR2 of X and Y human chromosomes, that *DDX11L16 *escapes from X inactivation and has an active homolog on the human Y chromosome (Figure 2C).

### *DDX11L *genes are present in multiple copies in primates' genomes

Recent comparisons among human and other primates genome, have revealed the presence of evolutionarily conserved sequences – syntenic regions – usually related to functional portions of the genome, such as protein-coding genes as well as non-genic sequences, probably with regulatory and structural functions [22,23]. To investigate the conservation of *DDX11L *genes along the primate evolution, human sequences obtained from direct sequencing of each *DDX11L *gene copy (see "Genomic structure and localization of DDX11L genes" section) and from the public databases (NCBI), were aligned with the sequences derived from BLAST analysis on chimpanzee, rhesus and orangutan genomes, using ClustalW algorithm. The analysis revealed that the human DDX11L genes have a high degree of conservation along primates, showing up to 98% of sequence homology to subtelomeric sequences from chromosomes XI and XIV of chimpanzee, and 91% with the subtelomeric sequences from chromosome 13 of rhesus. No DDX11L homologous were found in the orangutan genome assembly. Both in chimpanzee and in rhesus, the *DDX11L *homologous genes revealed to overlap – as already shown in the humans – a gene homologous to the human *WASH *in the PAR2 of the X chromosome.

In addition, the human *DDX11L *gene family members showed a significant sequence similarity with the *DDX11 *homologous gene of chimpanzee and rhesus on chromosomes IIp and 11, respectively.

**Figure 3:**
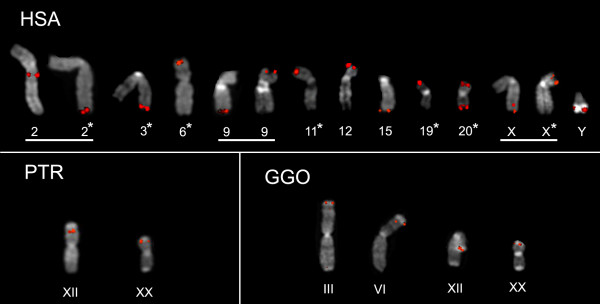
**FISH panel of HSA, GGO e PTR chromosomes**. Partial metaphases showing the chromosomal localization of the human DDX11L1 gene and of its orthologs on human, chimpanzee, and gorilla metaphases, determined by fluorescence in situ hybridization analysis. (A), (B) and (C) localization of the *DDX11L *specific probe on *Homo sapiens *(HSA), common chimpanzee, *Pan troglodytes *(PTR), and Gorilla gorilla (GGO) chromosomes, respectively. For an easy comparison of the results, the phylogenetic nomenclature was used. The asterisks indicate the *DDX11L *gene family members not present in the Human Genome Project final release (NCBI Build 36.1). Examples of polymorphic rearrangments detected on human chromosomes are underlined (2, 9, and X). Note that chimpanzee and gorilla have large blocks of very telomeric, complex heterochromatic sequences [39]. The DDX11L signals appear to map centromerically with respect to these blocks.

Since the subtelomeric regions of non-human primate genomes do not have a comprehensive coverage, there may be some non-human primate *DDX11L *gene copies that will be missing in this kind of analysis. Thus, to avoid this, and to better evaluate the presence of *DDX11L *genes along the primate lineage, also confirming the subtelomeric localization within human chromosomes, a comparative FISH analysis was performed against metaphase chromosomal spreads of *Homo sapiens *(HSA), chimpanzee (*Pan troglodytes*, PTR), gorilla (*Gorilla gorilla*, GGO), and orangutan (*Pongo pygmaeus*, PPY). A fragment of about 1,9 kb, corresponding to the exons 2 and 3 of *DDX11L*3, was amplified from a somatic hybrid cell line containing human chromosome 3 as the only human contribution, and was used as FISH probe (see Methods). Due to the highly polymorphic nature of the subtelomeric regions, the experiments were performed in metaphases from three different human individuals. When available (PTR and PPY), two distinct primate individuals were used. Examples of the FISH experiments are reported in Figure 3. Distinct hybridization signals were detected on the human chromosomes 2q13/14.1, 2qter, 3qter, 6pter, 9pter/9qter, 11pter, 12 pter, 15qter, 19pter, 20pter/20 qter, Xpter/Xqter, and Yqter. The 2q13/14.1 boundary corresponds to the region where the two short arm telomeres of the ancestral IIp and IIq acrocentric chromosomes fused to generate the human chromosome 2 [24]. Polymorphic rearrangements were detected on chromosomes 2, 9, and X. Thus, we cannot exclude that new intact copies of *DDX11L *genes might be found at other chromosomal ends in other individuals, since the subtelomeres undergo frequent inter-chromosomal sequence exchange [9].

Hybridization signals, using the same FISH probe, were also detected in the subtelomeres of chromosomes XII and XX of PTR, and in the subtelomeres of GGO chromosomes III, VI, XII, and XX (Figure 3), providing a relevant clue to the duplication events of large DNA patches occurred in the evolution of primates (see Discussion). No signals were detected in the orangutan metaphase chromosomes, confirming the *in silico *BLAST observation.

These data confirm the duplicated nature of the subtelomeric regions of human chromosomes, and clearly indicate that this gene emerged along the great apes lineage.

## Discussion

The subtelomeric regions of human chromosomes exhibit a dynamic nature and share wide regions of homology among them, providing an opportunity for non-homologous end joining (NHEJ) [9-11]. These regions are also involved in the attenuation of telomere position effect, demonstrated in yeast and some human cell lines [12,25,26]. In addition to a structural role, the subtelomeres of human chromosomes contain many gene families, mostly originated from duplication events [13,14].

The process of birth of a novel gene comprises initial mutational events – that give rise to new gene structure – followed by an evolutionary process in which the new gene structure becomes fixed in the species, and then it is improved for a new function [27]. Processes of exon shuffling (i.e. alternative splicing), retro-transposition, and gene duplication are the major mechanisms for generating novel genes, fixating the advantageous novelties. In many cases the emergence of a novel gene/function may be triggered for instance by some environmental variations [5,28].

We previously reported the presence of a pseudogene, CHL1p, within the subtelomeric region of the long arm of human X chromosome [29]. Since evidence has been found in Drosophila, in mouse, and recently in humans, of pseudogene functionality as well as of conservation [30-32], we were encouraged to investigate whether this region contained transcriptionally active sequences.

*The DDX11L *gene family members derive from a rearranged portion of the primate *DDX11 *gene (*alias CHLR1*, homologous to *CHL *helicase of *Saccaromyces Cerevisiae*), propagated among many subtelomeric locations (see table 1) as part of a segmental duplication [18]. The human subtelomeric *DDX11L *genes show up to 98,5% sequence identity with the exons 18 and 22–25, and the 3' UTR of *DDX11 *gene. The finding that *DDX11L *is a novel multicopy gene family present in different human and primate subtelomeres, and that all the identified gene copies undergo canonical splicing mechanism – and are also transcribed (at least in humans) – suggests this gene is emerging from an inactive pseudogene, and is probably undergoing a neo-functionalization process [18]. Therefore, this gene system may provide a valuable opportunity to investigate the emergence of a novel gene and a novel function in the recent human evolution.

Sequence comparison of the human *DDX11L *genes with the available databases, has shown the high degree of evolutionary conservation of this novel genes along the primates genomes, also revealing that these genes lie in proximity of *WASH *genes, within subtelomeres of many primates chromosomes.

Particularly, the *WASH *genes – pseudo and intact genes – were found in 16 different sites in human genome [14], and we have demonstrated that *DDX11L *and *WASH *genes co-localize within all described *loci*. *DDX11L *genes were found only in the primates, whereas *WASH *genes show orthologs in the vertebrates, flies, worms, slime mold, and entamoeba. The creation of a genomic block of about 8 kb, containing *DDX11L*-*WASH *genes (telomere to centromere orientation), occurred in the telomeres of a common ancestor of the humans and chimpanzee, in a period ranging from 65 to 8 million years ago (mya), after the evolutionary divergence of rodents and cat species from primates, and before the recombination event that created the PAR2 on the Y human chromosome. This multi-step hypothesis is strongly supported by the evidence that *DDX11L *emerged as novel gene family only in the primate genomes, and is completely absent in the wallaby, rodents and cat genomes [29]. Moreover, the presence of this genomic block on the primate X chromosome, and even on the Y chromosome of humans, strengthens this theory. The presence of the *DDX11L-WASH *block in the terminal regions of chromosomes in different individuals, provides clues about the history of its spreading through the genome, and throughout human populations.

**Figure 4:**
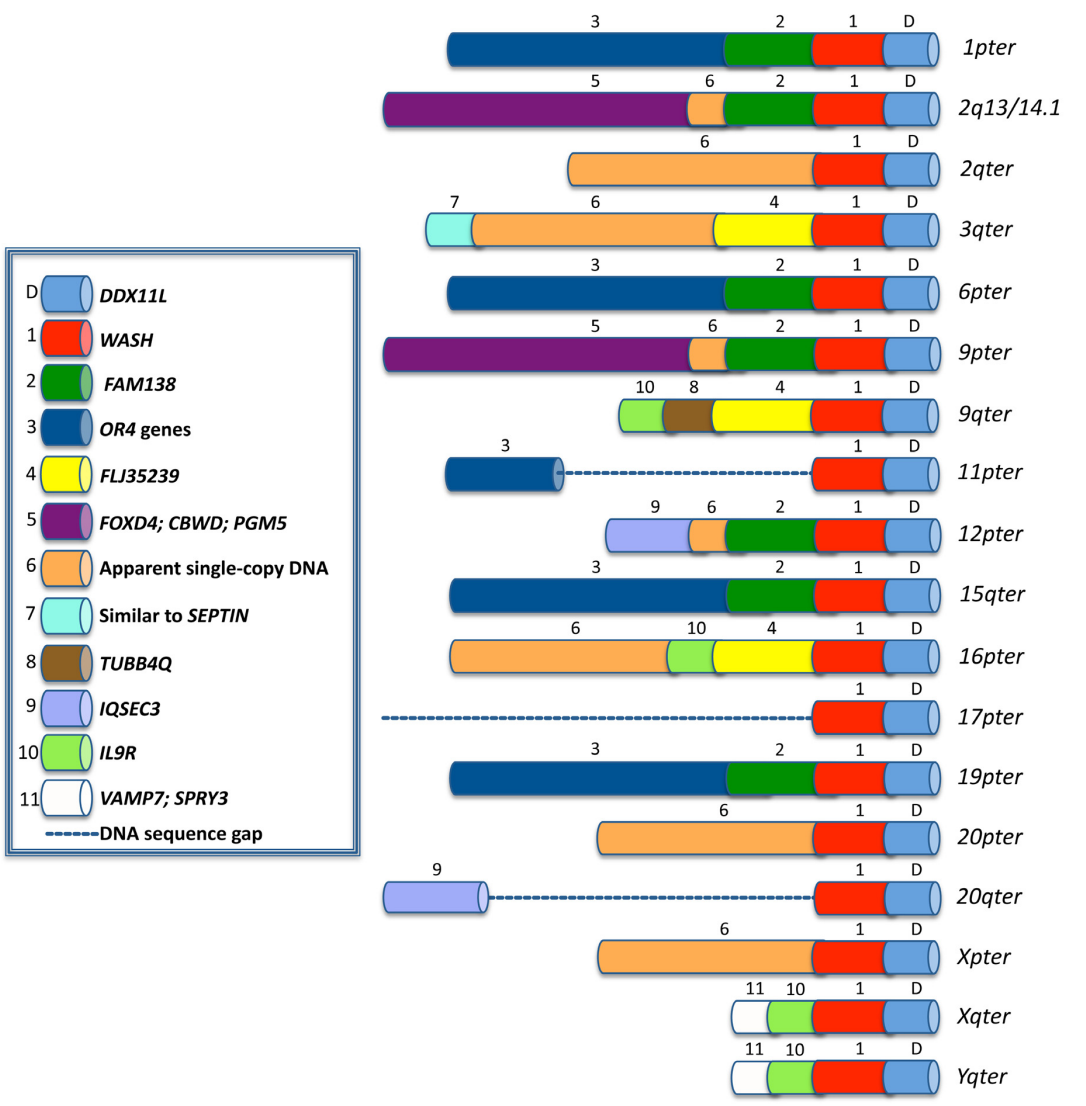
**Map of human subtelomeric sequence blocks**. Subtelomeric contigs are aligned at telomeres or to maximize alignments of paralogous blocks. Copies of a given block have the same colour and number (see legend in the figure). 2qFS_I represents one of the two ancestral telomeres fused head-to-head at 2q13-14; other internal paralogies are not shown here. (Modified by Linardopoulou et al., 2005)

*DDX11L *genes originated from an exon rearrangement of an ancestral *DDX11 *gene, that moved to the subtelomeric region of the long arm of an ancestral ape chromosome – adjacently to the *WASH *gene – from which originated the gene copy located on chromosome 12 [5,18,29]. The hypothesis is supported by the fact that all the sequenced *DDX11L *gene copies have the same telomere-to-centromere orientation, suggesting a common origin from an ancestral *DDX11L *copy. The presence of at least 18 *DDX11L *gene family members in the human chromosome pairs, compared to six copies on the PTR chromosomes (IIp, XI, XII, XIV, XX and on the sexual X chromosome), four in GGO (III, VI, XII and XX) and two in *Rhesus macaca *(13 and sexual X chromosome), provides a clear example of a multi-step evolutionary *scenario*, with the evidence that *DDX11L *gene has duplicated, and then moved, in different subtelomeric regions of human chromosomes in the time (about 8-5 mya) since chimpanzees and humans diverged. Subsequently, the major spreading of these sequences occurred throughout the human genome evolution (Figure 4). This process is still in progress, as these regions are highly polymorphic and show the highest rate of recombination overall the genome. Brown et al. [2] first discovered subtelomeric variability in the humans, demonstrating that some human subtelomeric block, analysed by FISH in more than one individual, showed variation in copy number and chromosomal location [2,9]. The human *DDX11L *genes are at high risk for genomic rearrangements, such as deletions or translocations. Thus, since we have detected polymorphic rearrangements by the analysis of only three unrelated human individuals, on three different chromosomes, we cannot exclude that other intact or partial copies of *DDX11L *genes might be identified within telomeres of other individuals.

Because of *DDX11L *proximity to the TAR (about 400 bp), it is clear that the telomere length, as well as the somatic variation in telomere organization, could greatly affect the expression of *DDX11L *genes. On the other hand, as hypothesized by Linardopoulou et al. (2005), subtelomeric dynamics might give a contribution to the normal human phenotypic variation and, more generally, to the diversification of these gene families [14].

Although we have identified at least 3 potentially translated *DDX11L *ORFs, with putative different aminoacid sequence and function, we cannot exclude that the transcripts may exert an unknown regulatory function. It has been shown that the genes encoding evolutionarily conserved protein, duplicated in multiple sites along primate genomes, might contribute to interspecies phenotypic differences [14]. The subtelomeric gene dosage changes, and the rapid genetic shuffling within these regions, may have important evolutionary consequences [8,33,34]. For instance, the telomeres maintenance pathways, mostly influenced by the recombination, could be affected by differential subtelomeric structure, sequence organization and copy number variation [35].

## Conclusion

Our data provide an additional clue to the duplication and the evolution of the human subtelomeres, confirming the high degree of plasticity of these regions, continuously involved in processes of genomic rearrangements and novel gene creation. The identification of a novel gene family emerging from human subtelomeric regions, and the evidence that an ancestral DNA patch has duplicated and then moved through the primates genomes in different ways and times, provide useful resources for a better understanding of the subtelomeres dynamics. However, additional targeted efforts are necessary to examine in depth the subtelomeric regions, in order to gain a complete understanding of the subtelomere evolution and functioning. Moreover, since their localization within highly dynamic human subtelomeric regions greatly predisposes these genes to rearrangements (duplications, deletions), *DDX11L *may contribute to normal human variation as well as to pathology.

Further detailed characterization of the *DDX11L *protein function(s), and an extensive genetic population analysis, will be needed to rule out the occurrence and the frequency of variant subtelomeric alleles, which could have advantageous, as well as detrimental or pathological, consequences on human health.

## Methods

### Similarity searches, EST alignments and protein prediction

BLAST searches in dbEST, assembly of ESTs and editing of consensus sequence was performed using Autoassembler program (ABI). Genomic similarity searches were performed at . All sequence alignments were performed using ClustalW algorithm. Exon prediction from genomic sequences was confirmed using AceView Gene, GeneScan and Vega Pseudogenes at Genome Browser web site . The analysis of coding sequences was performed using ORF Finder program . Protein sequence similarity searches were performed at NCBI against non-redundant protein databases.

### Sequence analysis

Sequencing of the IMAGE cDNA clones, PCR and RT-PCR products were performed using the dye terminator chemistry (Big Dye Terminator Cycle Sequencing II kit, Applied Biosystem, Foster City, CA, USA) according to the user manual instructions and analysed using an automated sequencer (ABI 3100; Applied Biosystem, Foster City, CA).

### Cell culture

Hamster/mouse-human somatic cell hybrids, containing each a different human chromosome, were cultured in DMEM/F12. For a list of somatic cell hybrids used in the present work, see Additional file 1.

### PCR and RT-PCR analysis

For semi-quantitative RT-PCR, total RNA from eight human tissues (brain, liver, skeletal muscle, heart, kidney, stomach, breast and colon) was purchased from Clontech. Total RNA from an RPE cell line, ARPE-19, lymphoblastoid (LCL), Y79 and teratocarcinoma cell lines were isolated by RNAzol B (Campro Scientific) and treated with Dnase I (Gibco/BRL). Semiquantitative RT-PCRs were performed as described [36]. A 5 μg aliquot of tissue RNA, isolated as previous described [37], was used for reverse transcription carried out using random hexanucleotide primers and SUPERSCRIPT III (Gibco BRL) in a 20 μl reaction according to provided protocol. PCR with DDX11L-specific primers was performed using 1 μl of the reverse transcription reaction as template in either a standard PCR reaction set-up with AmpliTaq Gold (Perkin Elmer). In each experiment, a sample without reverse transcriptase was amplified under the same conditions as the reverse-transcribed RNA. PCR products were purified from agarose gels by the QIAGEN Gel extraction Kit and directly sequenced on an automated sequencer using the ABI-PRISM big-dye terminator cycle sequencing ready reaction kit (Applied Biosystem). Oligonucleotide primers used in RT-PCR experiments were CHLRTF (5'-TTC TGG CCC CTG TTG TCT GC-3'), CHL1F (5'-GGG AAA GAT TGG AGG AAA GAT-3'), CHL1R (5'-ATT TCT CAC TGC CTT TTG TCT G-3'), CHL2F (5'-AGT TCA CTC CTG CCT TTT CCT T-3'), CHL3F (5'-CTT GCC GTC AGC CTT TTC TTT G-3'), CHL3R (5'-ACT GAC CCC GAC ACG TTT GCA T-3').

### FISH analysis

The genomic fragment of 1837 bp, used as FISH probe, was amplified from somatic hybrid cell line containing human chromosome 3 as the only human contribution, using the oligonucleotide pair CHL3F (described above) and CHLS1R (5'-TCC GTG AGA TCT TCC CAG GG-3'). We used this primer pair in order to cover a genomic region with the highest sequence similarity among human autosomes. Metaphase preparations were obtained from lymphoblastoid or fibroblast cell lines of the following species: human (*Homo sapiens*, HSA), common chimpanzee (*Pan troglodytes*, PTR), gorilla (*Gorilla gorilla*, GGO), Borneo orangutan (*Pongo pygmaeus pygmaeus*, PPY). Three different human individuals were examined. FISH experiments were performed essentially as described by Lichter et al. [38] with minor modifications. Digital images were obtained using a Leica DMRXA2 epifluorescence microscope equipped with a cooled CCD camera (Princeton Instruments, NJ, USA). Cy3 and DAPI fluorescence signals, detected with specific filters, were recorded separately as gray-scale images. Pseudocoloring and merging of images were performed using Adobe Photoshop software.

## Authors' contributions

VC, ACa, MR and ACi designed the experimental plan. VC, FG, MRR, MDE, MR and ACi analyzed the results. VC, ACa and RR performed the experiments. VC, FG, MRR, MDE, MR and ACi wrote the manuscript. FG, MRR, MDE, MD, MR critically read the manuscript. All authors have read and approved the final manuscript.

## Supplementary Material

Additional file 1 **Somatic hybrid cell lines**. The data provided represent a list of the monochromosomal hybrid cell lines, and the corresponding human chromosome they contain, used in this work. Moreover, a control gene that we tested for each chromosome is listed, in order to account the presence of the human chromosome in these cell lines. Click here for file
