# Supplementary material for: DDX11L: a novel transcript family emerging from human subtelomeric regions
Source: BMC Genomics. 2009 May 28;10:250. doi: 10.1186/1471-2164-10-250 (PMC2705379; doi:10.1186/1471-2164-10-250)
Supplement: Additional file 1 — Somatic hybrid cell lines. The data provided represent a list of the monochromosomal hybrid cell lines, and the corresponding human chromosome they contain, used in this work. Moreover, a control gene that we tested for each chromosome is listed, in order to account the presence of the human chromosome in these cell lines. [file 1471-2164-10-250-S1.doc]

Additional file 1

| **Hybrid cell line** | **Human Chromosome** | **Specific-gene** |
| --- | --- | --- |
| GM13139 | 1 | HPRP3 |
| GM10826B | 2 | MPP4 |
| GM10253A | 3 | PPARG |
| GM10115A | 4 | PDE6B |
| GM10114 | 5 | PDE6A |
| GM11580 | 6 | COL19 |
| GM10791A | 7 | RP10 |
| GM10156C | 8 | RP1 |
| GM10611A | 9 | TRIM32 |
| GM10926D | 10 | COL17A1 |
| GM10927B | 11 | MYOVIIa |
| Y.E210TC | 12 | GAPDH |
| RJ.387.58T1 | 13 | GJB2 |
| GM10479A | 14+16p | NRL |
| GM11418 | 15 | OCA2 |
| RJ.83.1FT16 | 16 | LUC7L |
| HY.137K | 17 | FSCN2 |
| GM11010A | 18 | STARD6 |
| GM10449A | 19 | CRX |
| GM13140 | 20 | MMP9 |
| WAV17 | 21 | WRB |
| GM10888A | 22 | GPR24 |
| HY.136C | X_a_ | SYBL1 |
| THX | X_i_ | XIST |
| GM06317A | Y | SYBL1 |
